# Supplementary material for: Heartbeat perception is causally linked to frontal delta oscillations
Source: Commun Biol. 2025 Oct 14;8:1466. doi: 10.1038/s42003-025-08933-9 (PMC12521737; doi:10.1038/s42003-025-08933-9)
Supplement: Supplementary file 4 — Reporting Summary [file 42003_2025_8933_MOESM4_ESM.pdf]

nature portfolio

Corresponding author(s): Prof. Dr. med Surjo Soekadar

Last updated by author(s): Aug 31, 2025

Reporting Summary

Nature Portfolio wishes to improve the reproducibility of the work that we publish. This form provides structure for consistency and transparency in reporting. For further information on Nature Portfolio policies, see our Editorial Policies and the Editorial Policy Checklist.

Statistics

For all statistical analyses, confirm that the following items are present in the figure legend, table legend, main text, or Methods section.

n/a	Confirmed	
☐	☒	The exact sample size (*n*) for each experimental group/condition, given as a discrete number and unit of measurement
☐	☒	A statement on whether measurements were taken from distinct samples or whether the same sample was measured repeatedly
☐	☒	The statistical test(s) used AND whether they are one- or two-sided<br>*Only common tests should be described solely by name; describe more complex techniques in the Methods section.*
☒	☐	A description of all covariates tested
☐	☒	A description of any assumptions or corrections, such as tests of normality and adjustment for multiple comparisons
☐	☒	A full description of the statistical parameters including central tendency (e.g. means) or other basic estimates (e.g. regression coefficient) AND variation (e.g. standard deviation) or associated estimates of uncertainty (e.g. confidence intervals)
☐	☒	For null hypothesis testing, the test statistic (e.g. *F*, *t*, *r*) with confidence intervals, effect sizes, degrees of freedom and *P* value noted<br>*Give P values as exact values whenever suitable.*
☒	☐	For Bayesian analysis, information on the choice of priors and Markov chain Monte Carlo settings
☒	☐	For hierarchical and complex designs, identification of the appropriate level for tests and full reporting of outcomes
☐	☒	Estimates of effect sizes (e.g. Cohen's *d*, Pearson's *r*), indicating how they were calculated

Our web collection on statistics for biologists contains articles on many of the points above.

Software and code

Policy information about availability of computer code

Data collection	A custom Simulink Real-Time model was used to adapt electric stimulation to the heartbeat in real-time. Bittium NeurOne EEG collection software was used to record EEG.
Data analysis	Python software using the publicly available MNE-Python (https://github.com/mne-tools/mne-python) and CLAM-NIBS (https://github.com/davidhaslacher/clam-nibs) packages was used to evaluate the data. A previously published algorithm (SASS) was used to assess EEG in the presence of electric stimulation artifacts (https://github.com/davidhaslacher/sass). Any scripts will be made available upon request.

For manuscripts utilizing custom algorithms or software that are central to the research but not yet described in published literature, software must be made available to editors and reviewers. We strongly encourage code deposition in a community repository (e.g. GitHub). See the Nature Portfolio guidelines for submitting code & software for further information.

Data

Policy information about availability of data

All manuscripts must include a data availability statement. This statement should provide the following information, where applicable:

- Accession codes, unique identifiers, or web links for publicly available datasets
- A description of any restrictions on data availability
- For clinical datasets or third party data, please ensure that the statement adheres to our policy

The raw EEG and behavioral data underlying this study are publicly available on G-Node (https://gin.g-node.org/davidhaslacher/commsbio-heartbeat-perception).

April 2023

Research involving human participants, their data, or biological material

Policy information about studies with human participants or human data. See also policy information about sex, gender (identity/presentation), and sexual orientation and race, ethnicity and racism.

Reporting on sex and gender	While we assessed participants' gender via self-report, and included an equivalent amount of male (14) and female (11) participants, we did not hypothesize any gender-specific differences in heartbeat perception. Therefore, all reported analyses were aggregated across genders.
Reporting on race, ethnicity, or other socially relevant groupings	No racial, ethnic, or other socially relevant groupings were considered in this work.
Population characteristics	In total, 25 participants (14 female, 11 male, 26 ± 5 years of age) were invited to participate in the study.
Recruitment	Participants were recruited through university job boards, and therefore reflect an educated young adult sample.
Ethics oversight	This study was approved by the ethics committee of the Charité – Universitätsmedizin Berlin (EA1/077/18).

Note that full information on the approval of the study protocol must also be provided in the manuscript.

Field-specific reporting

Please select the one below that is the best fit for your research. If you are not sure, read the appropriate sections before making your selection.

☒ Life sciences ☐ Behavioural & social sciences ☐ Ecological, evolutionary & environmental sciences

For a reference copy of the document with all sections, see nature.com/documents/nr-reporting-summary-flat.pdf

Life sciences study design

All studies must disclose on these points even when the disclosure is negative.

Sample size	We did not have robust assumptions about the relevant parameters (i.e. effect size) before collecting the data, severely limiting the conclusions about type I/II errors that can be drawn from such an analysis. Thus, the sample size was chosen to match that of a prior study on the effects of AM-tACS on perception [1].<br><br>[1] Haslacher, David, et al. "Working memory enhancement using real-time phase-tuned transcranial alternating current stimulation." Brain Stimulation 17.4 (2024): 850-859.
Data exclusions	One participant was excluded due to exceedingly high heartbeat detection accuracy (> 90%) in all conditions.
Replication	We did not attempt to replicate the effects described in this work yet, but have ensured detailed documentation of the methods to support independent replication.
Randomization	In this repeated-measures design, condition order was pseudorandomized within each participant.
Blinding	The threshold for AM-tACS was adjusted individually for each participant to the maximal level that remained below the threshold for somatosensory perception. Thus, participants were blind to the experimental condition (timing of AM-tACS relative to heartbeat).

Reporting for specific materials, systems and methods

We require information from authors about some types of materials, experimental systems and methods used in many studies. Here, indicate whether each material, system or method listed is relevant to your study. If you are not sure if a list item applies to your research, read the appropriate section before selecting a response.

Materials & experimental systems

n/a	Involved in the study
☒	☐ Antibodies
☒	☐ Eukaryotic cell lines
☒	☐ Palaeontology and archaeology
☒	☐ Animals and other organisms
☒	☐ Clinical data
☒	☐ Dual use research of concern
☒	☐ Plants

Methods

n/a	Involved in the study
☒	☐ ChIP-seq
☒	☐ Flow cytometry
☒	☐ MRI-based neuroimaging

Plants

Seed stocks	n/a
Novel plant genotypes	n/a
Authentication	n/a
